# Dr. Macqueen's Reply

**Published:** 1913-04-12

**Authors:** J. Gordon Macqueen


*To the Editor of* The Hospital.

### Dr. Macqueen's Reply.

*To the Editor of* The Hospital.

Sir,—There is an old saying, with much truth in it, that two swallows do not make a summer; nor, I take it, can two birds of passage change sordid atmospheric conditions which have existed for countless years into those of a more congenial clime.

To prate and prattle is an easy thing, but to disprove facts is an altogether different matter.

In The Hospital of March 15 last Dr. Shepherd and I revealed numerous facts regarding the condition of affairs which existed in the York County Hospital during the term of his and my own residency there. From various quarters since there have been attempts at denial, but, Sir, on the face of it, it is preposterous to think that two medical men should write a series of such grave accusations, giving the dates of their happening, in some cases even the exact minute, if they were such entire falsehoods as some have tried to make out. A feeling of resentment naturally grows in the breasts of those against whom the accusations are made, and attempts at self-justification are only to be expected.

I should have thought that the writer of the above letter would have preferred to have remained quiet, especially on the subject about which she has written—viz., the treatment of the nursing staff. She states that the charges have been thoroughly dealt with. I, personally, was not aware of the fact, but may I ask her if that was the reason of her resignation being handed in? And may I also ask her if she knows to whom the accusation "that in one of the wards a sister, losing control of herself, threw a book at a nurse in front of the patients," applies; or again to whom refers the accusation "that some of the nurses objected to being placed in certain wards on account of the treatment meted out to them by sisters?"

May I correct a misconception that seems to be afloat regarding the medical treatment of the nursing staff? The complaint was not that the sick nurses were not seen by the residents, but that there was in several cases a failure to report immediately to *any* medical man.

I may say that neither my colleague nor myself read the report of Sir Henry Burdett on the York County Hospital, published in The Hospital of January 27, 1912, until towards the end of 1912. Had I read the report of such an eminent hospital critic previously I, for one, would certainly never have thought of entering upon any appointment in the York County Hospital. When I did read it I entirely agreed with the statements made therein. Let the writer of the preceding letter read this carefully and remember that people in glass-houses should not throw stones—nor even books.—I am, Sir, yours faithfully, J. Gordon Macqueen.

### Co-operation Once More.

*To the Editor of* The Hospital.

